# Stress loading history of earthquake faults influenced by fault/shear zone geometry and Coulomb pre-stress

**DOI:** 10.1038/s41598-020-69681-w

**Published:** 2020-07-29

**Authors:** Claudia Sgambato, Joanna Phoebe Faure Walker, Zoë Keiki Mildon, Gerald Patrick Roberts

**Affiliations:** 1 Institute for Risk and Disaster Reduction, University College London, Gower Street, London, WC1E 6BT UK; 2 School of Geography, Earth and Environmental Sciences, University of Plymouth, Drake Circus, Plymouth, PL4 8AA UK; 3 Department of Earth and Planetary Sciences, Birkbeck, University of London, Malet Street, London, WC1E 7HX UK

**Keywords:** Natural hazards, Solid Earth sciences

## Abstract

Whether the stress-loading of faults to failure in earthquakes appears to be random or to an extent explainable, given constraints on fault/shear-zone interaction and the build-up and release of stress over many earthquake cycles, is a key question for seismic hazard assessment. Here we investigate earthquake recurrence for a system of 25 active normal faults arranged predominantly along strike from each other, allowing us to isolate the effects of stress-loading due to regional strain versus across- and along-strike fault interaction. We calculate stress changes over 6 centuries due to interseismic loading and 25 > Mw 5.5 earthquakes. Where only one fault exists across strike, stress-loading is dominated by the regional tectonics through slip on underlying shear zones and fault planes have spatially smooth stress with predominantly time-dependent stress increase. Conversely, where faults are stress-loaded by across-strike fault interactions, fault planes have more irregular stress patterns and interaction-influenced stress loading histories. Stress-loading to failure in earthquakes is not the same for all faults and is dependent on the geometry of the fault/shear-zone system.

## Introduction

The stress-loading of faults to failure in earthquakes is driven by regional tectonics, but is also influenced by fault interaction during earthquakes, evidenced by calculations of Coulomb stress transfer (CST) and corresponding changes in the rate of seismicity^1–3^. Faults also interact over longer time periods evidenced by fault displacement profiles that show steep displacement gradients and enhanced displacements on adjacent fault tips^4,5^, and observations of finite fault displacement profiles that adhere to global scaling relationships between fault length and fault displacement, both for isolated faults and for closely-spaced fault networks (*d* = γ*L*, where γ = 0.03 for both isolated faults and summed across the strike of fault networks^6,7^). However, despite the above evidence for organisation of both the stress-loading to failure process and long-term displacement accumulation, earthquake recurrence is often considered to be a random Poisson process for some seismic hazard purposes^8,9^. The link between fault interaction during single earthquakes and over multiple seismic cycles described above argues that the recurrence of earthquakes must sum to produce the long-term displacement profiles. Hence displacement accumulation during earthquakes over the long-term is not random, and must be influenced by interaction, which is in turn governed by fault system geometry. However, study of geometry-controlled interaction is complicated by the fact that fault systems are complex, commonly exhibiting multiple overlapping faults, and it is difficult to isolate the effects of earthquake-inducing stress accumulation caused by tectonic loading as opposed to loading due to rupture of neighbouring faults both along and across strike^10^. Without isolation of these effects it is challenging to assess what aspects of the stress-loading to failure, and hence seismicity, might be in some way explainable given observations of fault system geometries. Furthermore, stress-loading due to slip on shear zones in the lower crust, which have been observed in metamorphic rocks, and inferred to exist beneath active faults in the brittle crust, are rarely included in studies of interaction for brittle faults in the upper crust^10^. Hence, the effect of stress loading from shear-zones slip in the interaction process merits further study.

**Figure 1:**
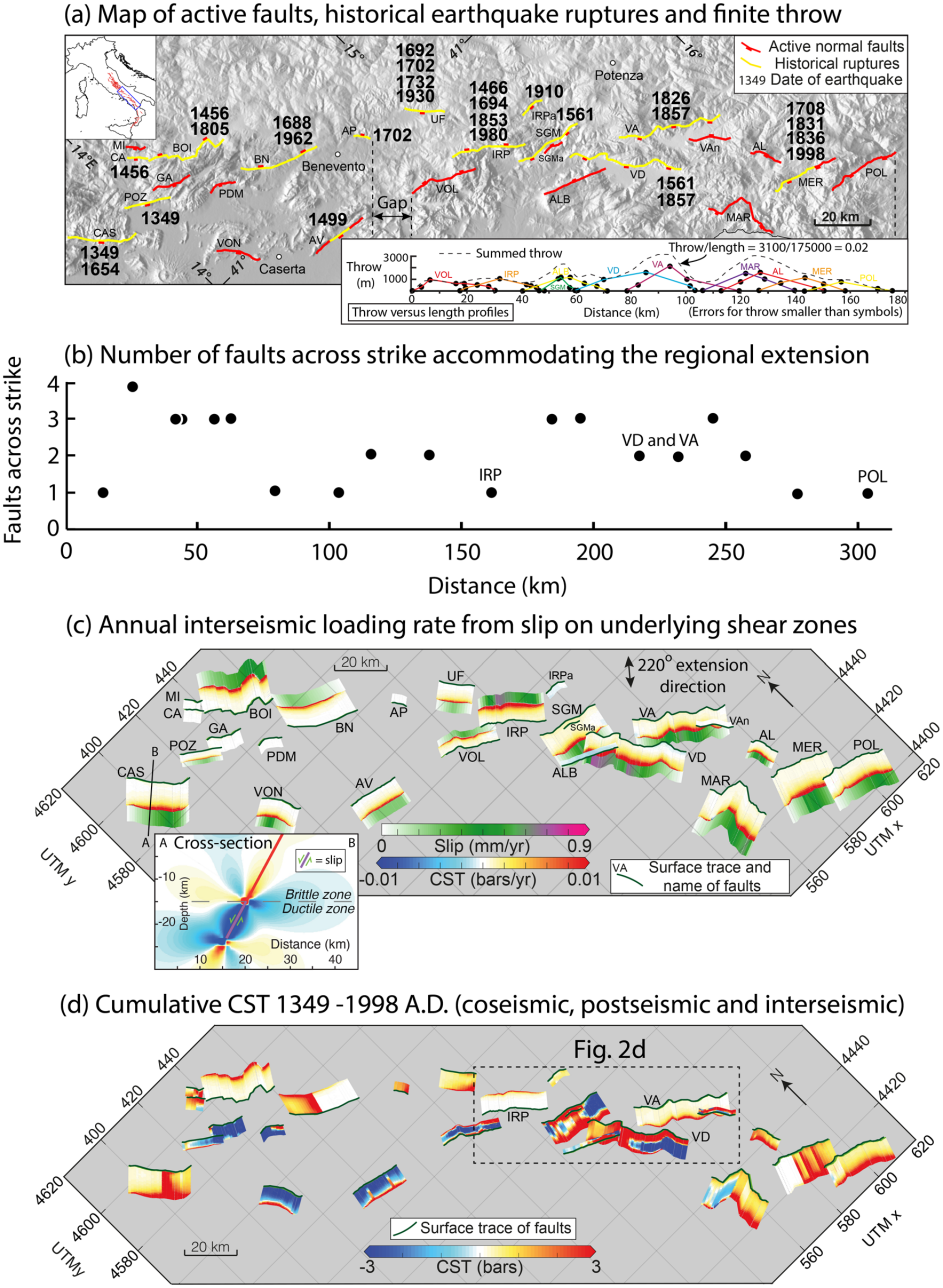
Characterisation of the study area. (**a**) Map of active faults in the Southern Apennines: fault traces are in red; faults rupturing in historical time with date of earthquakes are in yellow. Main towns are shown. Inset shows scaling relationships for the fault system in the area (data from reference^47^). (**b**) Plot showing the number of active faults across strike along the Southern Apennines. (**c**) 3D strike-variable active faults in the Southern Apennines, stress represents one year of interseismic loading rate transferred from shear zones to the brittle portion; both stress-loading onto the brittle portion of the fault and the annual slip distribution on the underlying shear zones we used is shown. (**d**) Map of cumulative coseismic, postseismic and interseismic CST for the timeframe considered in this study (from 1349 A.D. to 1998 A.D.). Dashed box includes the location of the area shown in detail in Fig. 2d. UTM coordinates are indicated in thousands of meters.

In this paper we examine a fault system in southern Italy where we can isolate the effects of regional tectonic loading from across strike fault interaction, because in places only a single fault accommodates all of the regional deformation, whereas elsewhere several faults exist across strike and interact to share the tectonic strain (Fig. 1). Furthermore, for this area the existence and behaviour of shear-zones is constrained by a study of the link between the topographic drive for extension and strain-rates measured over multiple millennia^11^. Viscous shear-zones in the lower crust drive slip on the overlying brittle faults, evidenced by a power-law relationship between slip-rates on upper crustal faults and topographic driving stresses, with an exponent 3.3 (range of 2.7–3.4 across at the 95% confidence level for length scales 5–60 km)^11^, that is reminiscent of that for viscous flow laws^12^. With the role of shear zones relatively-well constrained, we conduct an analysis of CST in the southern Apennines, Italy, that includes coseismic stress loading due to interactions between faults during earthquakes (25 > Mw 5.5 earthquakes since 1349 A.D.), plus interseismic loading from shear zone slip that includes postseismic afterslip. We search for aspects of the stress loading history that can be linked to fault system geometry and show that some aspects of the stress-loading to failure in earthquakes appears not to be random, but explainable given quantitative constraints on the fault interaction.

## Earthquake deformation in the southern Apennines and model set-up

The Southern Apennines are characterised by extension since 2–3 Ma^13–15^, associated with damaging earthquakes of Mw 5.5–7.1, occurring on active normal faults orientated NW–SE, such as the 1980 Irpinia earthquake, Mw 6.8, that caused 3,000 deaths, and the 1857 Val d’Agri earthquake, Mw 7.1, that caused upwards of 10,000 deaths^16^ (Fig. 1). Decadal extension rates measured with GPS and those implied by the measured offsets of slopes formed during the demise of the Last Glacial Maximum (15 ± 3 kyrs B.P.) across fault scarps are in reasonable agreement (~ 1–2 mm/year)^17–19^. This suggests that active normal faulting in this region is localised across a narrow fault system that in places has only a single fault accommodating the regional extension (Fig. 1b). The narrow zone in which extension occurs is confirmed by GPS data that show SW–NE velocities increase by ~ 1–2 mm/year across a zone only 10–20 km wide coincident with the position of the mapped active faults^17^. Throw across the faults has been measured from geological cross-sections, with individual faults having throw/length ratios of 0.03–0.06, with a value of 0.02 for the summed throw profile, similar to global values^18^ (Fig. 1a). The Italian historical earthquake catalogue for this region is considered to be complete for events with Mw > 5.8 after 1349 A.D.^16,20^. Attempts have been made to explain the historical sequence of earthquakes in the Southern Apennines through CST, which shows that most of the ruptures occurred in areas of high static stress increase produced by coseismic stress changes^21^. Since then, approaches to modelling Coulomb stress have improved, allowing inclusion of information on variable fault geometry^22^ and interseismic stress loading from underlying shear zones^10,23,24^. Thus, we have included these in our calculations.

**Figure 2:**
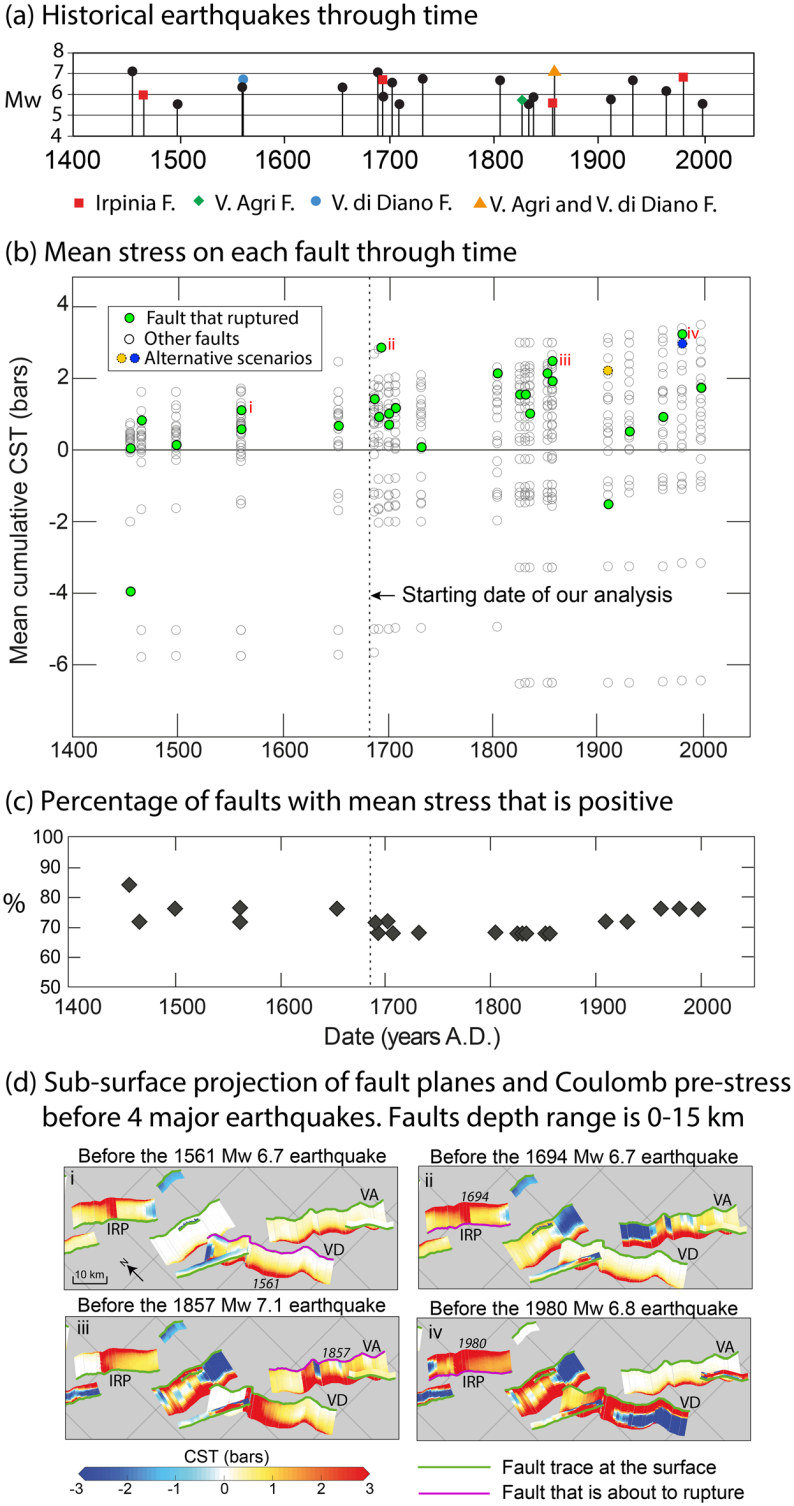
Analysis of cumulative CST and pre-stress heterogeneities prior to historical ruptures. (**a**) Historical earthquakes occurring since 1349 A.D., with coloured dots showing earthquakes occurring on the Irpinia fault, Val d’Agri fault and Vallo di Diano fault. (**b**) Mean cumulative CST calculated for all the faults prior to each historical earthquake. The faults that rupture (green circles) show positive mean cumulative CST since 1692 A.D., except for the 1910 earthquake, that occurred on the Irpinia Antithetic fault; yellow and blue circles represent alternative scenarios. (**c**) Plot showing the percentage of faults in the Southern Apennines that have mean Coulomb stress that is positive. (**d**) Panels showing the pattern of Coulomb pre-stress before some of the strongest earthquakes in the Southern Apennines. Location of the area is shown in the dashed box in Fig. 1d. The fault that ruptures next is indicated by purple lines. The figure highlights how the stress on the Irpinia fault before the events in 1694 and 1980 is relatively uniform (ii, iv), compared to the stress on the Vallo di Diano and Val d’Agri faults prior to the earthquakes in 1561 and 1857 (i, iii).

**Figure 3:**
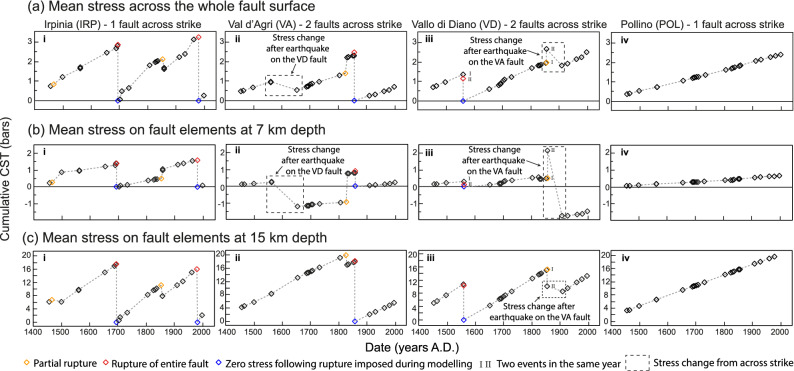
Comparison of Coulomb pre-stress over time. (**a**) Mean Coulomb pre-stress across the whole fault surface; (**b**) mean pre-stress calculated at 7 km depth; (**c**) mean pre-stress calculated at 15 km depth. Orange diamonds indicate partial ruptures, red diamonds are total ruptures of the whole fault surface; blue diamonds represent the imposed condition of zero stress on fault, immediately after a total rupture. (i) Coulomb pre-stress on the Irpinia fault. No stress changes due to ruptures on neighbouring faults are observed. (ii) Coulomb pre-stress on the Val d’Agri fault. Dashed boxes highlight stress changes due to rupture occurring on the Vallo di Diano fault, located across strike; at 15 km depth, the stress changes due to across-strike interaction are overwhelmed by the stress changes due to interseismic loading produced by slip on shear zones at depth; (iii) Coulomb pre-stress on the Vallo di Diano fault. Events marked as I–II refer to two events occurring in the same year, two events in July and August 1561, and two events separated by a few minutes in 1857. Dashed boxes highlight stress changes due to rupture occurring across strike on the Val d’Agri fault. (iv) Coulomb pre-stress for the Pollino fault. No known M > 5.5 earthquake is attributed to the fault in historical time.

We input the strike-variable geometry of the faults using published code^22^, modelled CST for each historical earthquake, and added interseismic loading from creep on underlying shear zones^10^ (Fig. 1c,d). Creep rates were assumed to be identical to slip-rates measured at the surface since 15 ± 3 ka^11^, with values from published sources and our own mapping^10,14,18,19,25–27^. Any postseismic afterslip on surface scarps would be included in surface slip values so the interseismic creep modelled includes postseismic afterslip, so we do not model postseismic stress changes due to viscoelastic afterslip separately^28^. Based on other studies, we hypothesise that the magnitude of stress changes due to distributed postseismic relaxation would be relatively small (typically ±  ~ 0.01 bars in 100 years)^29^, and thus overwhelmed by the ±  ~ 0.01 bars annual interseismic loading rate (Fig. 1c), given that the earthquakes we study are tens to hundreds of years apart in time. With the fault spacing we examine (typically ~ 15 km), post-seismic relaxation after earthquakes raises the stress both in the hangingwall and footwall of a Mw 6.9 earthquake by < 0.1–0.5 bars over tens of years at hypocentral depths, and along-strike post-seismic stress changes are also positive, but with even lower values^30^. Thus, post-seismic relaxation would, if included, modify coseismic CST changes by <  + 0.1–0.5 bars. Likely values would be mostly smaller for the example we study because only 3 of the 25 earthquakes we study have magnitudes of Mw 6.9 or more. Therefore, the magnitude of stress changes due to post seismic relaxation are likely to be similar in size or smaller than the symbols in Fig. 3, so this will not significantly change our results. The Coulomb stress ($\Delta CST)$ is defined as:

$$\Delta CST = \Delta \tau + \mu \left( {\Delta \sigma + \Delta P} \right)$$

where $\Delta \tau$ is the shear stress change, $\mu$ is the coefficient of friction, $\Delta \sigma$ is the change in normal stress and $\Delta P$ is the pore pressure change^31^. In this study we neglect pore fluid pressure changes, since we are interested to see if the stress-loading and seismicity can be interpreted without this variable^10^. We modelled cumulative CST for the time period containing 25 historical earthquakes on 16 active faults (from 1349 A.D to 1998 A.D., Fig. 2a,b; Supplementary Data1-Table 1). Although stress drops for large earthquakes are typically between 10–100 bars^32^, due to lack of information of how stress drop in these historical earthquakes relates to finite stress, we have imposed the condition where Coulomb stress is reset to zero on portions of faults that rupture. This is consistent with our aim of studying the temporal change in Coulomb stress, rather than absolute stress. The percentage of faults with mean positive CST (Fig. 2c), the maximum values for mean stress on individual faults, and mean stress values on individual faults (Fig. 2b) appear to be variable until the 1692 A.D. earthquake, when they appear to stabilise. Thus, we only analyse the data after that time, allowing the model a period of time to burn-in after the initial conditions of zero stress imposed on all the faults in 1349 A.D. Modelling results are shown in Figs. 1 and 2 and are compiled into two videos (Supplementary Video 1 and 2) showing the accumulation of CST over time.

## Observations of Coulomb stress transfer

Stress accumulation through time is spatially heterogeneous across the fault system and across individual faults (Fig. 1d; Supplementary Video 1–2), but some clear patterns emerge from our modelling.

Firstly, since 1692 A.D., 16 out of 17 of the earthquakes occurred on faults that had positive CST values before each earthquake (23 out of 25 earthquakes when considering the whole sequence, since 1456 A.D.; Supplementary Data 1c). The mean CST value on faults that ruptured was > 0.5 bars in 81% of the cases (Supplementary Video 1; Supplementary Data 1c), higher than the hypothesised CST triggering threshold of 0.1–0.5 bars^1,33,34^. A negative CST value is present only for the Mw 5.76 earthquake in 1910 A.D. that we have modelled on the Irpinia Antithetic fault, but there is uncertainty relating to the source responsible for this event; if the rupture occurred instead on the southern section of the Irpinia main fault, the mean CST is positive (2.19 bars, this alternative is shown with a yellow circle in Fig. 2b). Consequently, the pre-stress on the Irpinia fault prior to the Mw 6.8 earthquake in 1980 would be 2.92 bars (blue dot in Fig. 2b), 0.3 bar lower than it would be in the alternative scenario. Thus, after 1692 A.D., using the cumulative CST values produced by summed coseismic, postseismic and interseismic CST, all the earthquakes may have occurred on faults with positive mean CST values, even though up to ~ 30% of the faults had negative mean CST values. Note that if only coseismic stresses are considered, Coulomb stress values are − 4.0 to 1.5 bars, with 8 of the earthquakes occurring on faults with negative values, and with no obvious spatial pattern to the earthquake recurrence (Supplementary Video 2; Supplementary Data 1b). Hence studying the cumulative CST appears to provide a better explanation of the historical sequence than studying coseismic CST alone, in agreement with previous work in the central Apennines^10^.

Secondly, the 1694 A.D. Mw 6.73 earthquake occurred on the Irpinia fault which had the highest mean CST pre-stress (2.83 bars) of all the faults at that time (Fig. 2b,ii), with second highest value of 3.22 bars for the 1980 Mw 6.81 earthquake on the Irpinia fault (Fig. 2b,iv), exceeded at that time only by the value for the Monte Alpi fault (3.38 bars). The Irpinia fault is notable because it has no other known active faults across strike (Fig. 1b). Other earthquakes occurred on faults that were not the most positively-stressed at those times (Fig. 2b), and all those occurred on faults that have active faults across strike (Fig. 1b), or the earthquakes were < Mw 5.86 (e.g. the Mercure fault; Supplementary Data 1a).

**Figure 4:**
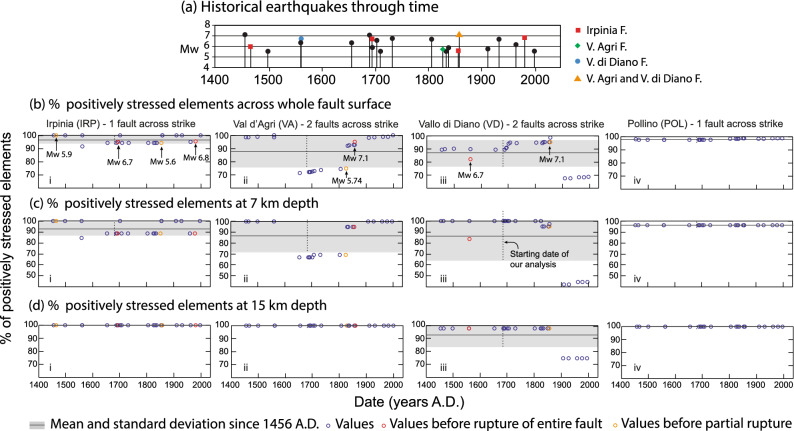
Analysis of Coulomb pre-stress heterogeneity on faults. (**a**) Magnitudes and timings of the historical earthquakes occurring since 1349 A.D.; coloured dots show earthquakes occurring on Irpinia fault, Val d’Agri fault and Vallo di Diano fault (note there were no known M > 5.5 earthquakes on the Pollino fault during the shown time period). (**b**) Percentage of positively stressed elements across the whole fault surface, calculated prior to each historical earthquake. (**c**) Percentage of positively stressed elements calculated at 7 km depth. (**d**) Percentage of positively stressed elements calculated at 15 km depth. Black lines represent the mean percentage over time; the grey area is one standard deviation. Orange circles indicate partial ruptures; red circles represent total ruptures of the whole fault surface. Where more than one fault exists across strike, the range of values for the percentage of positively stressed elements is wider.

Thus, to investigate if the presence or absence of another active fault(s) across strike has an effect on stress build-up and release we examine the progressive accumulation of pre-stress CST values before other major earthquakes (Mw > 5.86) for a variety of fault location geometries (e.g. 2 faults across strike; 1561, Mw 6.72, Vallo di Diano fault; 1857, Mw 7.12, Val d’Agri fault; 1 fault across strike; 1980, Mw 6.81, Irpinia fault; no historical earthquake, Pollino fault). We have excluded other large magnitude earthquakes if they occurred early in the time considered (e.g. 1456, Mw 7.1, Boiano fault), or if there is scarce information on the location and geometry of the fault that ruptured (e.g. 1688, Mw 7.06, possibly on the Benevento fault). In particular, we have compiled results that constrain the history of cumulative CST through time across the entire fault surfaces (Fig. 3ai–iv), and at different depths on the faults (Fig. 3bi–iv and Fig. 3ci–iv), and also the spatial distribution and percentage of positively and negatively stressed elements of the faults through time (Figs. 2d and 4). Since there is uncertainty relating to the depth of the seismogenic layer, alternate scenarios were explored (e.g. shear zone depth of 12–24 km rather than 15–24 km), but these showed no impact on the patterns of variations through time in the stress loading, except for a general increase in the mean stress across the faults.

The spatial distribution of positively and negatively stressed elements across faults at the time they rupture in earthquakes with Mw > 6.7 is complex, with patches of positive and negative stress concentrated at corrugations on the fault surfaces (evidenced by a change in strike at the surface scarp), and also in locations where there are neighbouring faults in close proximity (Fig. 2d).

In general, mean CST values across entire fault surfaces increase over time until they rupture in earthquakes (Fig. 3a). However, where only one fault exists across strike, the increase in Coulomb stress over time occurs at a relatively steady rate (Fig. 3ai,iv), dominated by the steady rate of loading from underlying shear zones, that mimics the steady rate of loading from tectonics and body forces^11^. In contrast, where more than one fault exists across strike, the increase through time is less steady, with decreases and increases in mean CST produced by earthquakes on neighbouring faults that enhance or counteract the effect of loading from underlying shear zones (see dashed boxes in Fig. 3aii,iii). A similar pattern emerges if the mean CST value is sampled only from narrow depth ranges (Fig. 3b,c).

In terms of the spatial variation in CST values across faults, earthquakes on the Irpinia, Val d’Agri and Vallo di Diano faults occurred when most of the fault surface was positively stressed (75–95%; Fig. 4b). However, there is a clear difference between faults with respect to the range and standard deviation for the percentage of elements with positive CST values when averaged over the time period 1456–1998 A.D., both over the whole fault surfaces (Fig. 4b), or over different depth portions of the fault surfaces (Fig. 4c,d). For the Irpinia fault, where only one fault exists across strike, 97 ± 3% of the elements over the whole fault surface have positive stress averaged over 1456–1998 A.D. (Fig. 4bi); the value is 98 ± 0%, for the Pollino fault which also has no other fault across strike (Fig. 4biv). In cases where two faults exist across strike, values are 88 ± 12% and 87 ± 10% (Val d’Agri and Vallo di Diano faults respectively). Similar patterns emerge for samples at different depths (Fig. 4c,d). Furthermore, the Irpinia fault had 95% of its whole fault surface positively stressed when it ruptured in both the Mw 6.73 1694 A.D and Mw 6.81 1980 A.D. earthquakes, whilst only 82% of the elements were positively stressed on the Vallo di Diano fault when it ruptured in Mw 6.72 1561 A.D. earthquake (although this was early in the model history), and 95% of the elements positively stressed in the Mw 7.12 1857 A.D. earthquake on the Val d’Agri fault. Partial rupture occurs with variable values for the percentage of elements with positive stress (75–95%; e.g. 75% for the Mw 5.74, 1826 A.D., Val d’Agri fault).

Furthermore, whilst the Irpinia and Pollino faults experienced a narrow range of values of 92–100% and 98% respectively of elements with positive CST at the times of historical earthquakes, the Val d’Agri and Vallo di Diano faults experienced a wider range, with values of 71–100% and 67–98% respectively (Fig. 4b). Similar observations can be made for the calculated percentage of positively stressed elements at 7 km depth (Fig. 4c). The range for the Irpinia fault is 88–100% and 96% for the Pollino fault; the Val d’Agri and Vallo di Diano exhibit a range of 67–100% and 42–100% respectively. However, looking at 15 km depth, the Irpinia, Val d’Agri and Pollino faults show a percentage of positively stressed elements that remain constant at 100% through time (Fig. 4d), due to the fact that the interseismic loading from a fault’s shear zone at depth overwhelms stress transfer between neighbouring fault/shear-zone structures at this depth. Only the Vallo di Diano fault shows a range of 74–98%, as a result of the effects of the Mw 7.1 1857 A.D. earthquake on the Val d’Agri fault.

Together, the results on the percentage of elements with positive CST values show that (1) CST values are spatially smooth where only one fault exists across strike with more spatial variation where two or more faults exist across strike, and (2) earthquakes can occur even when the entire fault surface has not accumulated positive CST, but the range of values when earthquakes occur appears to be smaller where only one fault exists across strike. Examination of CST through time shows that these observations, (1) and (2), are due to the proximity of fault/shear-zone structures across strike and the stress reductions they produce when the faults rupture in earthquakes (Fig. 2d; Supplementary Video 1). The effect of this is particularly clear where we have been able to isolate the effects of regional loading from underlying shear zones and local loading due to across-strike fault interaction, that is, for the case of the Irpinia fault where this structure is the only active fault across strike. As noted above, for two out of 25 historical events, both on the Irpinia fault, the fault that ruptured was characterised by essentially the highest CST pre-stress (Fig. 2b). We argue that this is due to the fact that there are no faults across strike to produce stress reductions and hence small patches of reduced stress are rare, and when they do form they only cover < 6% of the Irpinia fault (Figs. 2b, 3, 4). This observation may explain why the two earthquakes in 1694 and 1980 A.D. are known as so-called “twin events”, as they share overlapping damage distributions and similar magnitudes^35^, and show similar coseismic offsets in palaeoseismological trenches^36^. Our results show that they also share similar values for the mean cumulative CST when they occurred (~ 3 bars), both for the whole fault surface, and at specific depths (~ 1.5 bars at 7 km and ~ 16 bars at 15 km, Fig. 3), an equal percentage of fault surface positively stressed before the events (95% for both earthquakes; Fig. 4), and a similar values of maximum CST on an individual element of the fault surface, ~ 28.9 before the 1694 event and ~ 26.5 before the 1980 event (Supplementary Data 1c).

Overall, our results suggest that clear patterns emerge in the histories of stress-loading to failure in earthquakes for faults if cumulative CST is studied (summed coseismic, postseismic and interseismic stresses) and that CST is influenced by the geometry of the fault/shear-zone system.

## Discussion

In order to perform probabilistic forecasts of the propensity for particular faults to rupture in the future, and hence mitigate for the hazard, it is essential to include information on how faults behave and interact through time. Efforts on this topic have highlighted alternative views^9^ where earthquake recurrence is considered to be either a random Poisson process where there is no memory in the system with regard to previous earthquakes^8^, or a time-dependent process where the probability of occurrence of given earthquake magnitudes, and the slope of fault specific frequency-magnitude curves, are computed for each source fault using, for example, Brownian passage time or Weibull distributions^37–39^. Our study strongly supports the notion that there is a time-dependent process influenced by fault geometry, in that stress-loading to failure occurs through far-field tectonic loading and near-field interactions between faults. One key observation is that faults dominated by regional loading, without loading due to across-strike interaction, have stress-loading to failure histories that differ from those affected by across strike interaction. We highlight the question of whether it could be that the seismicity on such isolated faults differs from that on faults with across-strike interactions, and have presented some limited observations in support of this from the occurrence of so-called “twin earthquakes” on the Irpinia fault^35^. Whether these interactions produce differences in fault behaviour and seismicity may be tested with earthquake simulators that produce synthetic earthquake catalogues, based on modelling of fault interaction in complex fault networks^40^. However, a second key observation is that our results suggest that such simulations should include cumulative CST that, as has been achieved here, includes coseismic, postseismic and interseismic loading and the influence of underlying shear zones, not just slip on faults in the seismogenic layer. Moreover, our results show that it is important to choose appropriate samples of the depth variation in CST on faults in such simulations. We show that the base of the seismogenic layer (15 km in our models) appears to have stress-loading histories that are less affected by across strike interaction in the long-term, because stress increases quasi-linearly through time (Fig. 3), with less spatial variation in stress (Fig. 4d); this is because at that depth stress changes due to across-strike interaction are in some instances overwhelmed by stress changes due to interseismic loading produced by slip on shear zones at depth. In contrast, at a depth of 7 km stress-loading histories are more complex, with stress reduction due to across-strike interaction (Fig. 3), and a greater degree of spatial variation (Fig. 4c); at these depths stress reductions may serve as stress barriers that limit/delay rupture propagation^24,41^, and perhaps even control the propensity for the fault to rupture in a variety of earthquake magnitudes rather than a single so-called characteristic earthquake magnitude^42^. Overall, this study has isolated the effects of regional tectonic stress-loading and stress-loading due to across-strike fault interaction and shown that this approach provides new insights into how faults behave for studies of seismic hazard and continental deformation.

## Methods

### Modelling 3D strike-variable faults

Twenty-five faults in the Southern Apennines have been modelled with realistic variable geometries that were measured in the field, since it has been shown that CST is most sensitive to variations in strike of the receiver faults, compared to variations of dip and rake^22^. The method used to build strike-variable fault planes is described in detail in reference^22^. Fault traces, based on detailed fieldwork and Google Earth satellite imagery, are used to project the fault plane to depth. Faults are then modelled by discretising the fault plane in 1 km rectangular elements; the Coulomb stress changes are calculated for each element. The CST calculations are performed in the Coulomb 3.4 software^43^.

### Modelling historical coseismic and interseismic CST

We modelled 25 historical earthquakes, occurring on 16 faults, following the methodology described in reference^10^. We have included three earthquakes that occurred in Molise-North Campania, in the northern section of the study area, which have been previously modelled^10^. All the earthquakes are modelled with relatively simple, concentric slip distributions, since there is no detailed information for historical earthquakes. Thus, it is assumed that the coseismic slip is maximum in the centre of the fault and zero at the base of the seismogenic layer. To model the interseismic CST, shear zones are built from 15–24 km depth, as a continuation of the brittle portion of the faults. The depth chosen for the brittle/viscous transition is consistent with hypocentral depths and coseismic slip in the Apennines^44–46^, and consistent with previous modelling^10,11^. The annual rate of slip is calculated using the Holocene throws measured at surface, using new data and data from previous works^10,14,18,19,25–27^. A triangular profile is assumed for the interseismic slip distribution, by linearly interpolating between the values of slip-rate measured at the surface, and thereafter decreased to zero at the tips. The interseismic CST prior to each earthquake is calculated by multiplying the annual rate of slip and stress transfer by the number of years between two subsequent earthquakes.

### Calculating cumulative CST

The Italian catalogue of historical seismicity is considered complete since 1349 A.D.^20^. Thus, we start the model with zero stress prior to this date, since information on previous events are insufficient. To calculate the cumulative CST prior to each earthquake, the coseismic CST and interseismic CST are summed for each element constituting a fault plane. When an earthquake occurs, all the stress accumulated is released, so that the stress on the fault rupturing drops to zero. We allow the model to spin-up between 1349–1692 A.D., and do not analyse the data until after this time, to counteract the effect of our simplification where we assign CST as zero at 1349 A.D..

### Comparing cumulative CST and stress heterogeneity

To investigate the importance of geometry for stress heterogeneity, we compare the cumulative CST calculated prior to each historical earthquake for faults located across-strike from each other (VA, VD) and faults with no faults across strike (IRP, POL). The mean cumulative CST is calculated for the whole fault surface and at specific depths (7 km, 15 km), by calculating the mean stress for all the elements constituting 1 km of the fault surface in the downdip direction. To further investigate and compare the behaviour of across-strike and isolated faults, we calculated the percentage of positively stressed elements prior to each event both across the whole fault surface and at specific depths (7 km, 15 km).

## Supplementary information

Supplementary file1

Supplementary file2

Supplementary file3

Supplementary file4

## Data Availability

All data generated during this study are included in the Supplementary Data files.
