# Supplementary material for: Stress loading history of earthquake faults influenced by fault/shear zone geometry and Coulomb pre-stress
Source: Sci Rep. 2020 Jul 29;10:12724. doi: 10.1038/s41598-020-69681-w (PMC7391730; doi:10.1038/s41598-020-69681-w)
Supplement: Supplementary file 2 — Supplementary file2 [file 41598_2020_69681_MOESM2_ESM.docx]

**Stress loading history of earthquake faults influenced by fault/shear zone geometry and Coulomb pre-stress**

**Claudia Sgambato^1^*, Joanna Phoebe Faure Walker^1^, Zoë Keiki Mildon^2^, Gerald Patrick Roberts^3^**

^1^ Institute for Risk and Disaster Reduction, University College London, Gower Street, London, WC1E 6BT, UK

^2^ School of Geography, Earth and Environmental Sciences, University of Plymouth, Drake Circus, Plymouth PL4 8AA, UK

^3^ Department of Earth and Planetary Sciences, Birkbeck, University of London, Malet Street, London, WC1E 7HX, UK

* Corresponding author: e-mail address: [claudia.sgambato.17@ucl.ac.uk](mailto:claudia.sgambato.17@ucl.ac.uk)

**Description of Supplementary material**

File name: Supplementary Data 1

Description: Excel spreadsheet containing the following:

Table1: Field data used to calculate throw-rate and interseismic rate loading

a: Details of modelling for each historical earthquake

b: Mean coseismic Coulomb Stress Transfer on faults that rupture

c: Mean cumulative pre-stress on faults that rupture

d: Mean cumulative stress on all faults before each earthquake in the historical catalogue

e: Mean cumulative pre-stress across whole fault surface and at depth

f: Percentage of positively stressed elements through time

File name: Video 1

Description: Video showing the coseismic CST associated with 25 historical earthquakes from 1349 – 1998 A.D. in the Southern Apennines. The fault traces at the surface are shown in green, the trace of the fault that ruptures is shown in yellow, the trace of the next fault that ruptures is shown in pink.

File name: Video 2

Description: Video showing the cumulative CST (including interseismic and coseismic loading) prior to each historical earthquake from 1349 – 1998 A.D. in the Southern Apennines. The fault traces at the surface are shown in green, the trace of the fault that is about to rupture is shown in pink.
